# Floral anatomy and ultrastructure of *Lepanthes calodictyon*,* L*. *saltatrix* and* L*. *tentaculata* (Orchidaceae)

**DOI:** 10.1186/s12870-025-07141-1

**Published:** 2025-09-30

**Authors:** Max Rykaczewski, Małgorzata Kapusta, Magdalena Narajczyk, Dorota Łuszczek, Emilia Brzezicka, Dariusz L. Szlachetko

**Affiliations:** 1 Laboratory of Biologically Active Compounds, University of Gdansk, Intercollegiate Faculty of Biotechnology UG & MUG, Abrahama 58, Gdansk, 80-307 Poland; 2 Department of Plant Experimental Biology and Biotechnology, University of Gdansk, Wita Stwosza 59, Gdańsk, 80-308 Poland; 3 Laboratory of Electron Microscopy, University of Gdansk, Wita Stwosza 59, Gdansk, 80-308 Poland; 4 Department of Plant Taxonomy and Nature Conservation, University of Gdansk, Wita Stwosza 59, Gdansk, 80-308 Poland

**Keywords:** Micromorphology, Orchidaceae, Osmophore, Pleurothallidinae, Ultrastructure

## Abstract

**Background:**

Previous studies have shown that the ultrastructure and histochemistry of flowers give insight into the pollination mechanisms of orchid species. The microscopic features of species within the Pleurothallidine subtribe are poorly understood. In this study, we investigated three *Lepanthes* species: *L*. *calodictyon*, *L*. *saltatrix* and *L*. *tentaculata*. These species have a similar flower morphology that is distinct from that of the other representative of the genus.

**Results:**

Our analyses revealed the presence of features usually associated with myophily. The papillae of lips and petals of the *L*. *calodictyon* group were rich in lipids and proteins, which were correlated with high metabolic activity. Furthermore, the ultrastructural and morphological features were similar to those observed in other fragrance-emitting orchid species.

**Conclusions:**

Histochemical, ultrastructural and morphological features of the papillae on the surface of the lips and petals of examined taxa indicate that the papillae are osmophores.

## Introduction

The subtribe Pleurothallidinae Lindl. is one of the richest and most diverse groups among the Orchidaceae. It comprises approximately 40 well-recognised genera and over 5100 species [21, 25, 26, 35, 36, 44, 45]. Representatives of the Pleurothallidinae are usually small, mainly epiphytic, Neotropical orchids with a sympodial growth habit, and they predominantly occur at elevations above 1000 m in the humid rainforest or the páramo formation [25, 34–36, 44, 49]. Together with unrelated members of the *Bulbophyllum* genus, Pleurothallidinae species are considered as fly-pollinated orchids [6, 10, 15, 37]. The representatives of the Pleurothallidinae subtribe exhibit a large diversity of morphological characteristics associated with myophily. They usually have small brightly coloured flowers, and the surface is usually covered by papillae, motile hairs or trichomes, which attract pollinators. Nectary production and fragrance emission are regarded as factors that increase the effectiveness of pollination. However, among the approximately 40 recognised and acknowledged genera, only a few have been investigated thus far: *Acianthera* Scheidw. [7, 37], *Anathallis* Barb. Rodr. [7], *Dracula* Luer [17], *Lepanthes* [5], *Masdevallia* Ruiz & Pav. [7], *Octomeria* R.Br. [4], *Pleurothallis* R.Br. [14, 16], *Restrepia* Kunth, [46], *Scaphosepalum* Pfitzer [47], *Specklinia* Lindl. [9, 27], *Stelis* Sw. [1] and *Zootrophion* [7]. Nectary production has been demonstrated once [27], and most commonly, the presence of osmophores has been detected.

Among the Pleurothallidinae, *Lepanthes* Sw. is the most species-rich genus [26]. It comprises approximately 1200 species distributed from southern Florida, through Central America to Bolivia in the south. The greatest diversity has been observed in Ecuador and Colombia [34–36, 44, 49]. Species belonging to *Lepanthes* are characterised by the presence of dilated and ciliate sheaths on the ramicaul, which bear small, often yellow, flowers with petals that are broader than they are long. Typically, they have a trilobed lip with a midlobe reduced to a minute appendage. The blades of the lip are above the column, which is elongated and embraced by the lip. Although the appendage varies in form, it is most often covered by motile trichomes or hairs and is typically pressed beneath the column [34–36, 44, 49]. Moreover, the flowers are scentless (at least for humans) and do not offer a reward for pollinators, such as nectar or pseudonectar (e.g. [49]).

Sexual deception is considered by many authors as pollination syndrome in *Lepanthes* [15, 35, 44, 49, 60, 61]. The first observation of male flies mating with *Lepanthes* was recorded in Costa Rica by Blanco and Barboza [5]. They photographed and described males of *Bradysia floribunda* Mohrig (Sciaridae) that had attached their sexual organs to the appendage of the lip of *L*. *glicensteinii* Luer. The male flies of *B*. *floribunda* visited the *L*. *glicensteinii* flowers exclusively, suggesting very specific plant–pollinator interactions. Moreover, Blanco and Barboza [5] distinguished three crucial steps in pollinator behaviour. The first is the reception of the “olfactory” signal,flies hover around the plant for a few seconds, and after landing, they try to detect the scent produced by the flower. In the second step, when the flower is already open, the fungus gnats look for the female and start their mating dance. The last step is the reception of the sensory signal. Only after securing the appendage of the lip did the flies start to stake out the right position and “copulate” with the flowers. During pseudocopulation, pollinia are removed from the column and attached to the abdomen of the fungus gnats. The spermatophore is deposited on the ventral side of the column, near the appendage. The authors also reported that *B*. *floribunda* males pollinated the flowers [5].

*Lepanthes calodictyon* Hook., *L*. *tentaculata* Luer & Hirtz and *L*. *saltatrix* Luer & Hirtz form a group of species that are distinctive in both floral and vegetative traits when compared to other *Lepanthes* representatives. The species of this group are characterised by the simultaneous presence of papery reticulated leaves (vs. succulent non reticulated) with undulate margins (vs. glabrous), short and dense inflorescences (vs. usually long and lax) on the upper side of the leaf (vs. underside), bilobed petals contracted in to slender tails at the apex, unlobed or slightly bilobed lips without appendages (vs. distinctly trilobed with prominent appendage), and a lip hinged to the column by a claw (vs. sessile), which distinguish them from other *Lepanthes* species (differences are given in parentheses). The petals and lip are bright or deep red or crimson-cream and papillate in contrast to the inconspicuous, similar in shape, glabrous, pale yellow-green sepals. Compared to other *Lepanthes* species typical of high altitudes, species of the* L*. *calodictyon* group occur exclusively in humid lowland forests. Additionally, species of the *L*. *calodictyon* group are closely molecularly related and group together in a separate clade nested within the *Lepanthes* mega clade (Rykaczewski in prep.). Their unique morphology and distribution make these species interesting in a biological context; thus, they were investigated in detail here.

In Orchidaceae, vivid colours and various appendages on the surface of the floral segments are frequently connected to secretory activity. We suspect that the contrast red colouration of the petals and lip could implicate the crucial role of these floral segments in pollinator attraction. This hypothesis about the very particular role of the central parts of the flower seems legitimate, especially when considering the lack of a lip appendage. Therefore, the aims of this study were to investigate the morphological, histochemical and ultrastructural features of petals and the lip and to reveal the secretory activity of their cells using light, fluorescent and electron microscopy methods. Members of the genus *Lepanthes* have not previously been studied in terms of ultrastructure or histochemistry. This study represents the first report and expands our knowledge about micromorphology, ultrastructure and cytochemistry in Pleurothallidinae.

## Material and methods

### Plant source

Specimens of *L*. *calodictyon*, *L*. *saltatrix* and *L*. *tentaculata* were purchased from Ecuagenera Ltd. (Ecuador). Flowers were collected at anthesis from March 2016 to September 2017. The flowers of *L*. *calodictyon*, *L*. *saltatrix* and *L*. *tentaculata* (voucher numbers: UGDA 012573, 012605 and 012614 respectively) were preserved in KEW mixture (53% ethanol, 37% water, 5% formaldehyde and 5% glycerol) and deposited in the herbarium.

### Light microscopy

Plant materials were examined and measured using a SteREO Discovery V12 Zeiss stereomicroscope with a magnification range of 5 × to 63 × and an ocular graticule 10 ×/2.3. All measurements were collected according to the central axis of the structures. Photographs of the flowers were taken using a Zeiss Axiocam 512 and Nikon SMZ 1500 (Precoptic Co., Warsaw, Poland). The preliminary identification of active secretory regions of the flower was performed according to the method described by Wiśniewska et al. [65] and consisted of staining with a 0.01% solution of methylene blue or 0.1% neutral red.

Whole flowers and/or parts were used for light microscopy analyses and preserved in phosphate-buffered saline (PBS, pH 5.7) containing 2.5% glutaraldehyde and 4% paraformaldehyde at 4 °C in the dark. After 24 h, samples were rinsed twice in PBS, followed by distilled water. They were then dissected, dehydrated by increasing ethanol concentrations [23] and embedded in Steedman’s Wax (Sigma Aldrich, Poznań, Poland, [32]. The histochemical tests were performed on 10-µm sections made on HM 360 Microm and included staining with toluidine O for general histology [18], Coomasie Brilliant Blue (CBB) for protein detection [22], modified), Sudan Black B for lipids [8], and the periodic acid-Schiff reaction (PAS), which was used to determine the total insoluble polysaccharide content [24]. Catechol-type dihydroxyphenol inclusions were visualised using a 10% (w/v) aqueous FeCl_3_ solution [19]. Microtubules were visualised using rat primary antibody against α-tubulin (Ab6161,Abcam, Cambridge, UK and a goat anti-rat secondary antibody conjugated with DyLight™ 549 (AS12 2084,Agrisera, Vannas, Sweden. F-actin was detected using the monoclonal mouse antibody against actin (MP Biomedicals, ABO, Gdansk, Poland and goat anti-mouse secondary antibody conjugated with Alexa Fluor 488 (Life Technologies, Warsaw, Poland. Both primary and secondary antibodies were used in 1:800 dilutions. Nuclear chromatin was stained with 7 µL/mL DAPI (4′,6-Diamidino-2-phenylindole, Sigma-Aldrich, Poznań, Poland. In negative control experiments, the primary, secondary or both antibodies were omitted. All light microscopy sections were mounted in Mowiol medium and viewed using a Leica DM6000 B (Kawaska, Piaseczno, Poland or Nikon Eclipse E 800 (Precoptic Co., Warsaw, Poland).

### Electron microscopy

Samples for scanning electron microscopy were fixed overnight at room temperature in the KEW mixture, dehydrated through a graded ethanol series, coated with gold, and observed on a Philips XL-30 scanning electron microscope (Labsoft, Warsaw, Poland). Sample preparation for transmission electron microscopy (TEM) was performed using standard electron microscopy procedures [30], followed by post-fixation overnight in 1% OsO_4_. Plant materials were embedded in Spurr’s resin, cut to ultrathin sections and examined on a FEI Tecnai Spirit BioTWIN transmission electron microscope (Labsoft, Warsaw, Poland).

## Results

**Fig. 1:**
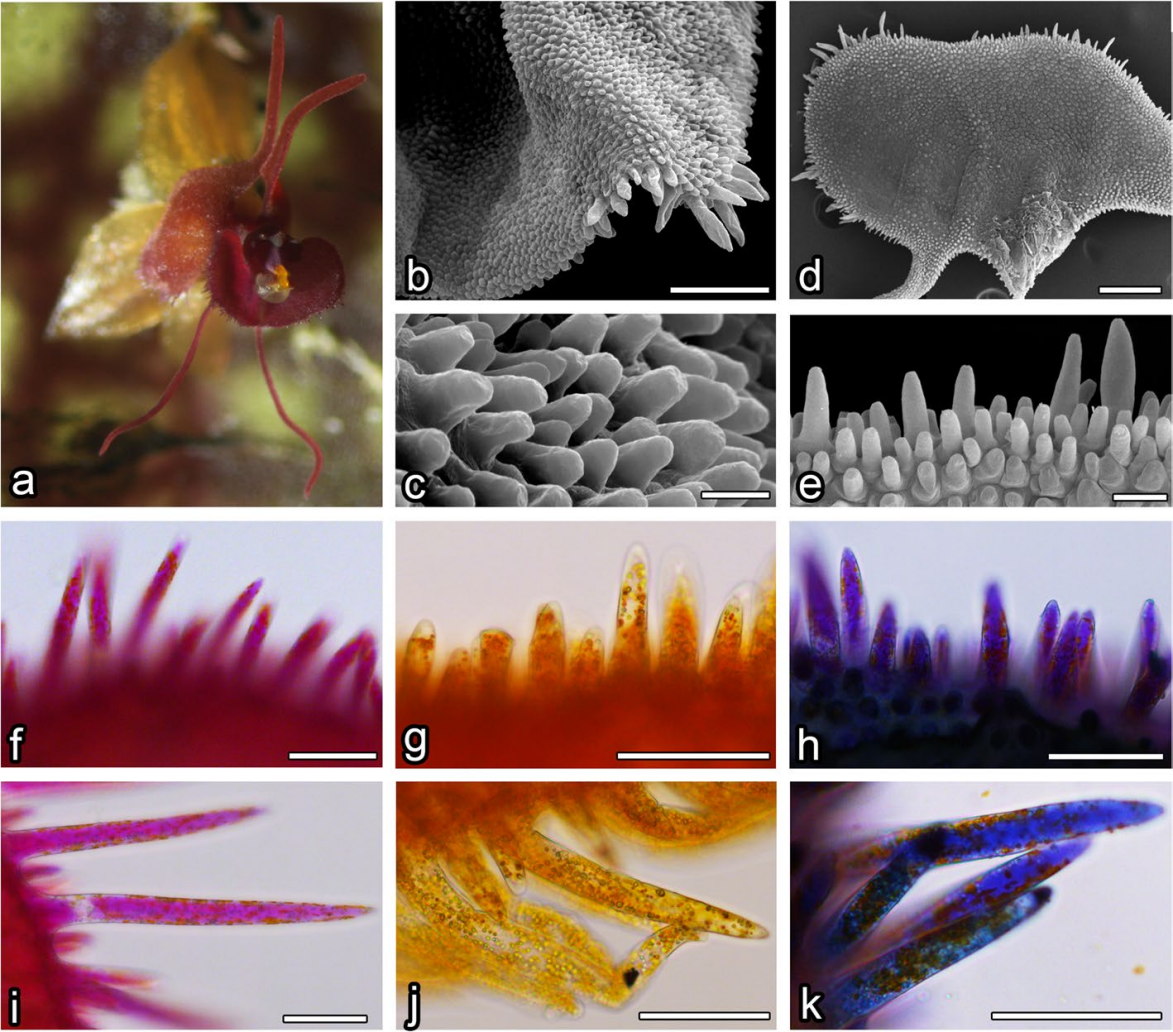
L. calodictyon. **a** General view of the flower. SEM images: (**b**, **c**) lip showing visible papillae, (**d**, **e**) petal with visible papillae. Results of staning with neutral red (**g**, **j**) and methylene blue (**h**, **k**) on papillae of lip (**f**–**h**) and petal (**i**-**k**), with comparison to unstained samples (**f**, **i**). Scale bars: **b** – 100 µm; **c** – 10 µm; **d** – 200 nm, **e** – 20 nm; **f**-**k** – 50 µm

**Fig. 2:**
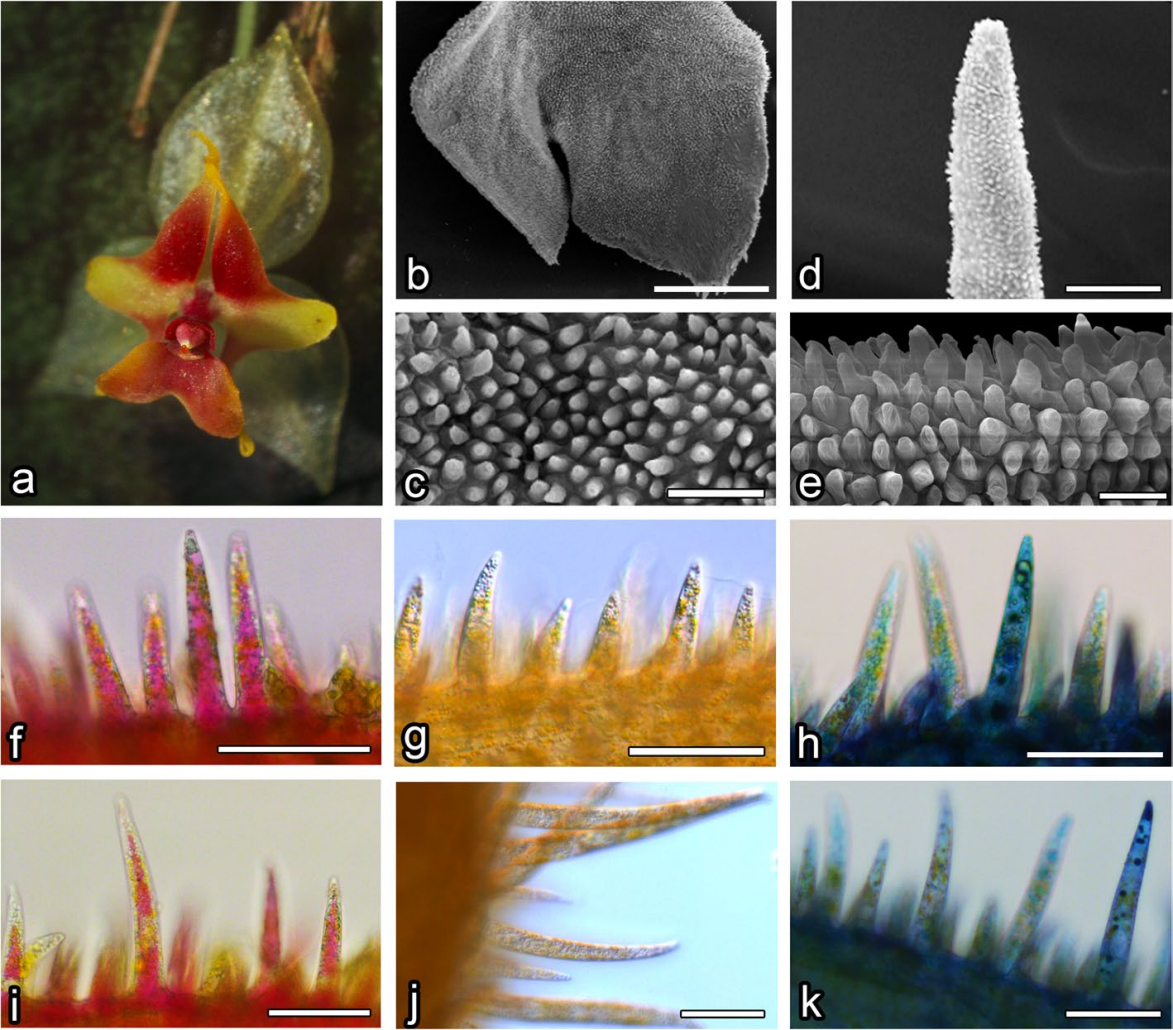
L. saltatrix. **a** flower overview; SEM results: **b**, **c** lip, visible papillae, **d**, **e** petal, visible papillae; results of neutral red (**g** and **j**) and methylene blue (**h** and **k**) staining of labellar (**f** – **h**) and petal’s papillae (**i**—**k**) with comparison to not stained ones (**f** and **i**). Bars: **b** – 500 µm; **c** – 50 nm; **d** – 50 nm, **e** – 20 nm; **f**-**k** – 50 µm

**Fig. 3:**
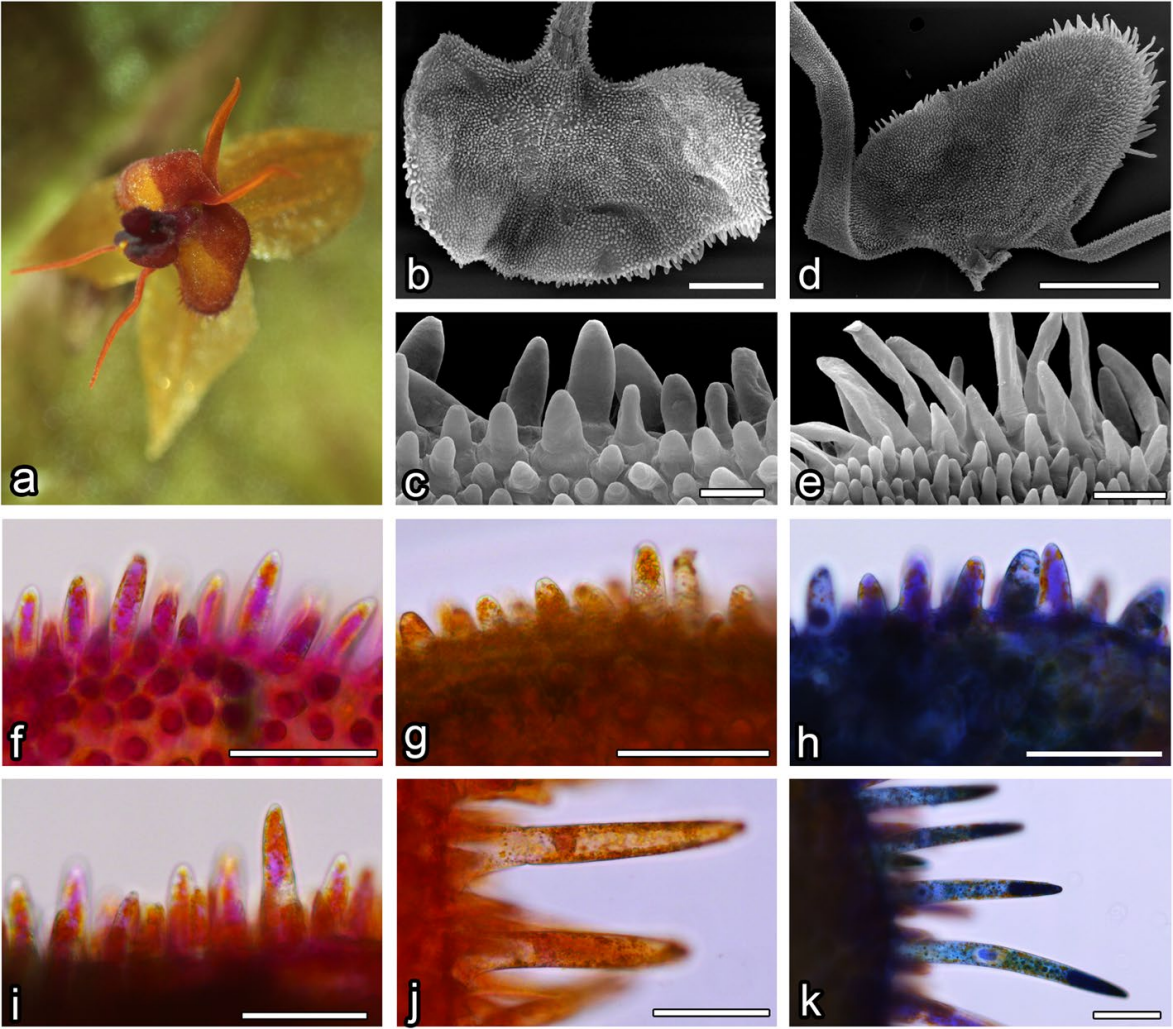
L. tentaculata. **a** flower overview; SEM results: **b**, **c** lip, visible papillae, **d**, **e** petal, visible papillae; results of neutral red (**g** and **j**) and methylene blue (**h** and **k**) staining of labellar (**f** – **h**) and petal’s papillae (**i** – **k**) with comparison to not stained ones (**f** and **i**). Bars: **b** – 200 µm; **c** – 20 µm; **d** – 500 nm, **e** – 50 nm; **f**-**k** – 50 µm

Among the three species (Figs. 1, 2 and 3), all flower segments, except the lip and petals, were similar in shape. The flowers did not produce any noticeable scent and emerged from the congested inflorescence on the top of the reticulated leaves. The data on the histochemistry and features of the flowers are summarised in Table 1.

**Table 1:** The results of micromorphological, ultrastructural and histochemical analyses of *Lepanthes calodictyon, L. saltatrix* and *L. tentaculata*

Species/method	*Lepanthes calodictyon*		*Lepanthes saltatrix*		*Lepanthes tentaculata*	
	petal	lip	petal	lip	petal	lip
Osmophores LM: methylene blue, neutral red	+	+	+	+	+	+
Secretions SEM	-	-	-	-	-	-
Proteins LM: CBB	+ + +	+/+ + +	+ + +	+ + +	+ + +	+ + +
Starch grains LM: PAS	-	-	-	-	-	-
Dihydroxyphenols In plastids/vacuoles LM: FeCl_3_	+	-	-	-	-	-
Lipids LM: SBB	+	+ + +	+ + +	+ + +	+ +	+ +
Secretions TEM	+	+/+ +	+	+ +	+	+ +
Cuticle LM: auramin 0	s	s	s	s	s	s
Cytoskeleton (α-tubulin and F-actin) LM	+ +	+ +	+ +	+ +	+ +	+ +

+ + +—abundant; + +—rich; +—present;—- absent; nt- not tested; s- smooth

### Petals

**Fig. 4:**
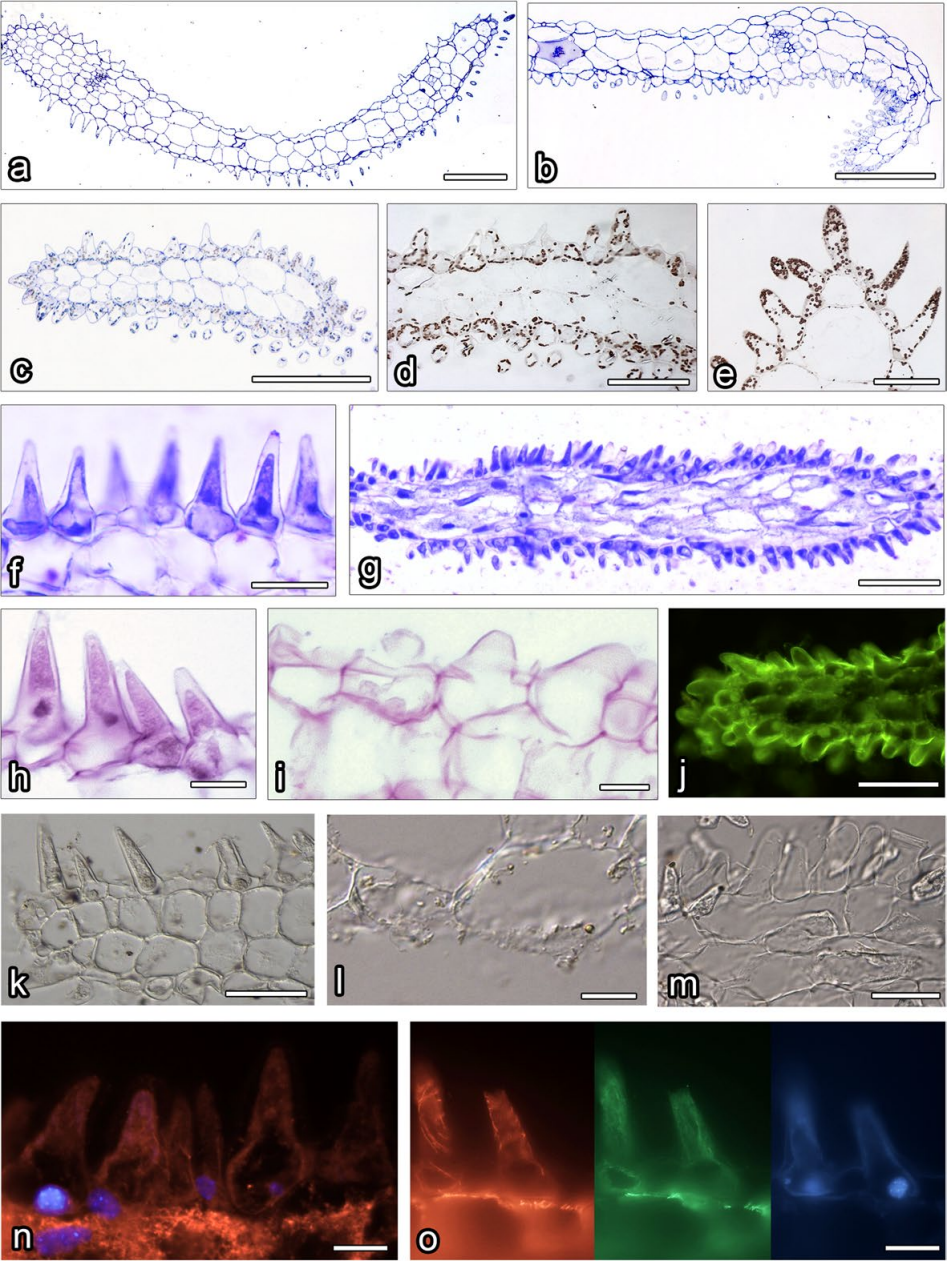
Histochemical features of petals of *L. calodictyon* (**c** and **d**, **g**, **j**, **l**, **n**), *L. saltatrix* (**a**, **e** and **f**, **h**, **k**, **o**) and *L. tentaculata* (**b**, **i**, **m**). **a**—**c** Transverse section (TBO), **d** and **e** abundant lipid droplets in trichomes (SBB), **f** and **g** lack of starch grains, **j** intact cuticle with no visible disruptions (Auramine O), **k**—**m** FeCl_3_ staining, dihydroxyphenol inclusions only visible in **l**, **n** – **o** cortical hoop like microtubules (red fluorescence), delicate microfilaments network (green fluorescence) and chromatin staining (blue fluorescence) in epidermal cells. Scale bars: **a**, **b**, and **c** – 100 µm; **d**, **g**, **j**, and **k**,—50 µm; **e**, **f**, and **m** – 20 µm; **h**, **l**, **n**, and **o** – 10 µm

**Fig. 5:**
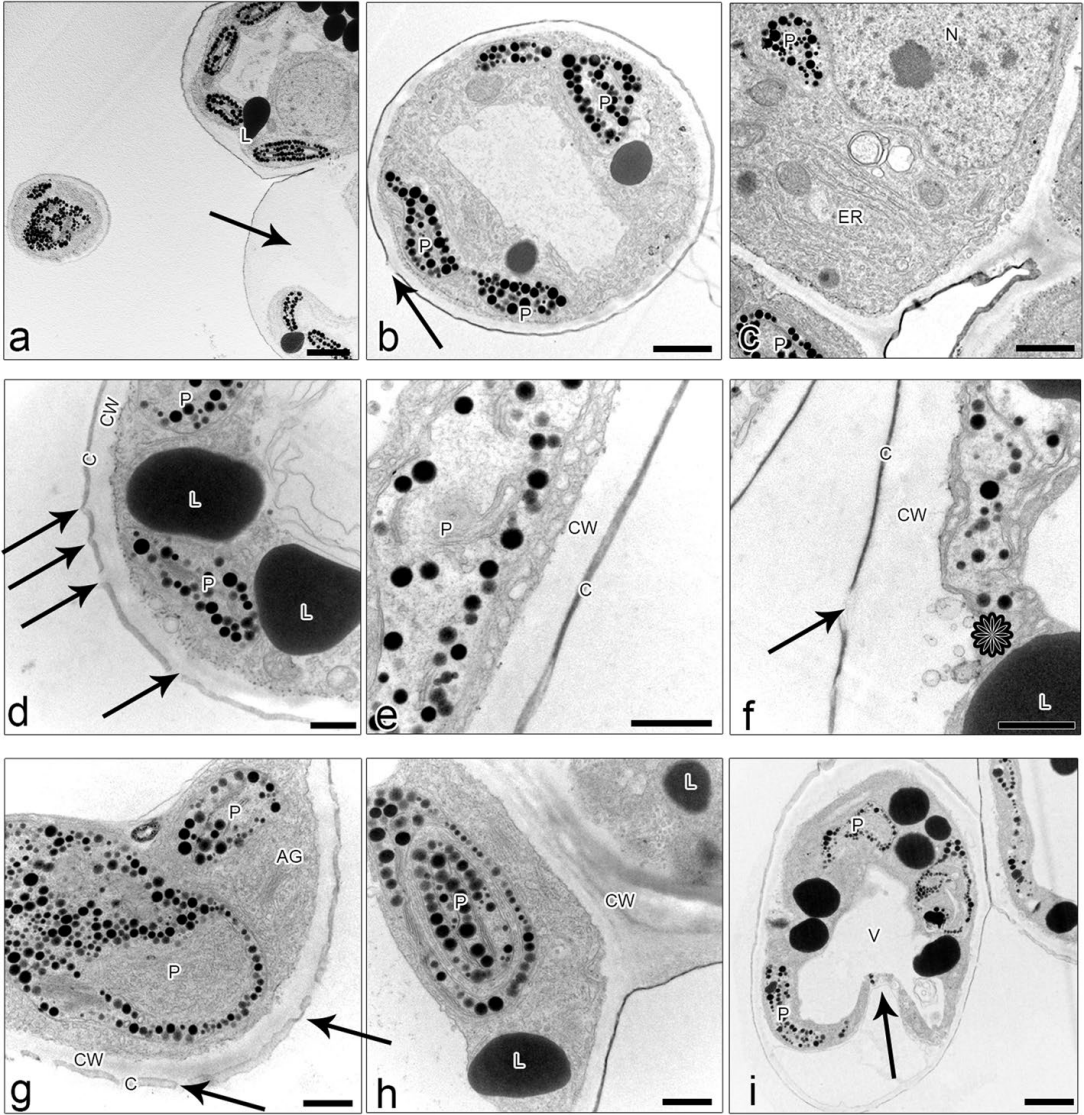
Ultrastructural features of *L. calodictyon* petals (**a** – **c**), *L. saltatrix* (**d** – **f**) and *L. tentaculata* (**g** – **i**). Epidermal cells with dense cytoplasm, numerous plastids with plastoglobuli, and internal membranes (**a** – **i**), profiles of ER (**c**, **h**), fully developed dictyosomes (**g**), lipid droplets (**a** and **b**, **d**, **f**, **h** and **i**). Cuticle with visible abruptions (**b**, **d**, **f**, **g**) and vesicles connected with plasmalemma (**f**, asteriks). Some epidermal cells with collapsed proplasts (**a**, **i**, arrow). c = cuticle, cw = cell wall, ER = endoplasmic reticulum, l = lipid droplets, n = nuclei, p = plastid, v = vesicles, v = vacuole. Scale bars: a – 2 µ; b, c and i – 1 µm; d-h – 500 nm

The petals were red in *L*. *calodictyon* and *L*. *tentaculata* and bicoloured in *L*. *saltatrix* (crimson on the upper lobe and cream on the lower lobe). The petals were bilobed with long, slender tails on both apices (Figs. 1, 2 and 3). The lobes of the petals and the tails on the apices were covered by unicellular papillae, similar in morphology to these observed on the surface of the lip (Figs. 1, 2 and 3, 4a–c). An intense colouration was observed in each species after treatment with neutral red and methylene blue was noticed (Figs. 1f–k, 2f–k, 3f–k). In all species, the cuticle of all examined petals was consistent when stained using Auramin O and lacked cracks (Fig. 4j). Ultrastructural studies revealed small abruptions of the cuticle on *L*. *saltatrix* and *L*. *tentaculata* (Fig. 5d, f–g), whereas no disruptions of the cuticle were observed in *L*. *calodictyon* (Fig. 5a).

The surface of the petals of *L*. *calodictyon* was covered by two cell types: regular, isometric epidermal cells and papillae cells (Fig. 1d, e). Bottle-shaped, unicellular papillae were formed by epidermal cells on the edge of the petal. The cuticle of the papillae was smooth without any secretions (Fig. 1e). Similar papillae were visible on the surface of the lengthened, thread-shaped petal. The middle part was built by regular epidermal cells with smooth cuticles. The petal was composed of two components: a rounded middle part and two lengthened, thread-like side parts.

In *L*. *saltatrix*, the surface of petal’s middle part was covered by bottle-shaped, unicellular papillae, which densely filled whole area (Fig. 2d). Similar papillae cells were visible across the whole surface of the side part. Their cuticle was smooth, but no secretions were observed (Fig. 2e).

Petals of *L*. *tentaculata* were composed of a rounded middle part and two lengthened, thread-like side parts. The surface of the petal’s middle part was covered by papillose cells (Fig. 3d). Bottle-shaped, unicellular papillae densely filled the entire area, and lengthened, unicellular trichomes were only located on the edge (Fig. 3e). Similar papillae cells were visible across the entire surface of the side part. Their cuticles were smooth, but no secretions were observed (Fig. 3e).

Histochemical studies revealed that petals’ papillae of the three investigated species were rich in proteins and lipids (Table 1; Fig. 5a–g). TEM revealed the presence of features associated with secretory activity. Residues of secretions were detected on the surface of petals’ papillae in *L*. *calodictyon* and *L*. *saltatrix*, but no secretions were found in* L*. *tentaculata* (Fig. 5a–i). The ultrastructure of the petals’ papillae vacuole occupied the main part of the cells, and the cytoplasm was dense with numerous organelles crowded on the perimeter of the cell (Fig. 5a–i). The small vesicles connected with plasmalemma were also observed between irregular plasmalemma and the cell wall (Fig. 5f).

SBB treatment indicated lipids deposited inside the cells, especially large amounts of lipid bodies in the trichomes of *L*. *saltatrix* (Figs. 4e and 5d). Lipid bodies were often accompanied by plastids (Fig. 5b, d, h, i). Within plastids, numerous osmiophilic plastoglobuli were observed, but no starch grains were noted (Fig. 5a–i). This observation is comparative with all examined species. A negative result was observed for the PAS reaction to detect starch grains (Fig. 4h,i). Few, small dihdroxyphenol inclusions were observed in the cytoplasm of *L*. *calodictyon* petals (Fig. 4l). Other staining methods remained inconclusive (Fig. 4k, m). The cytoskeleton of the petals’ papillae was well developed in all three species, and cortical hoop-like microtubules and a delicate network of microfilaments were noted (Figs. 4n–o).

### Lip

**Fig. 6:**
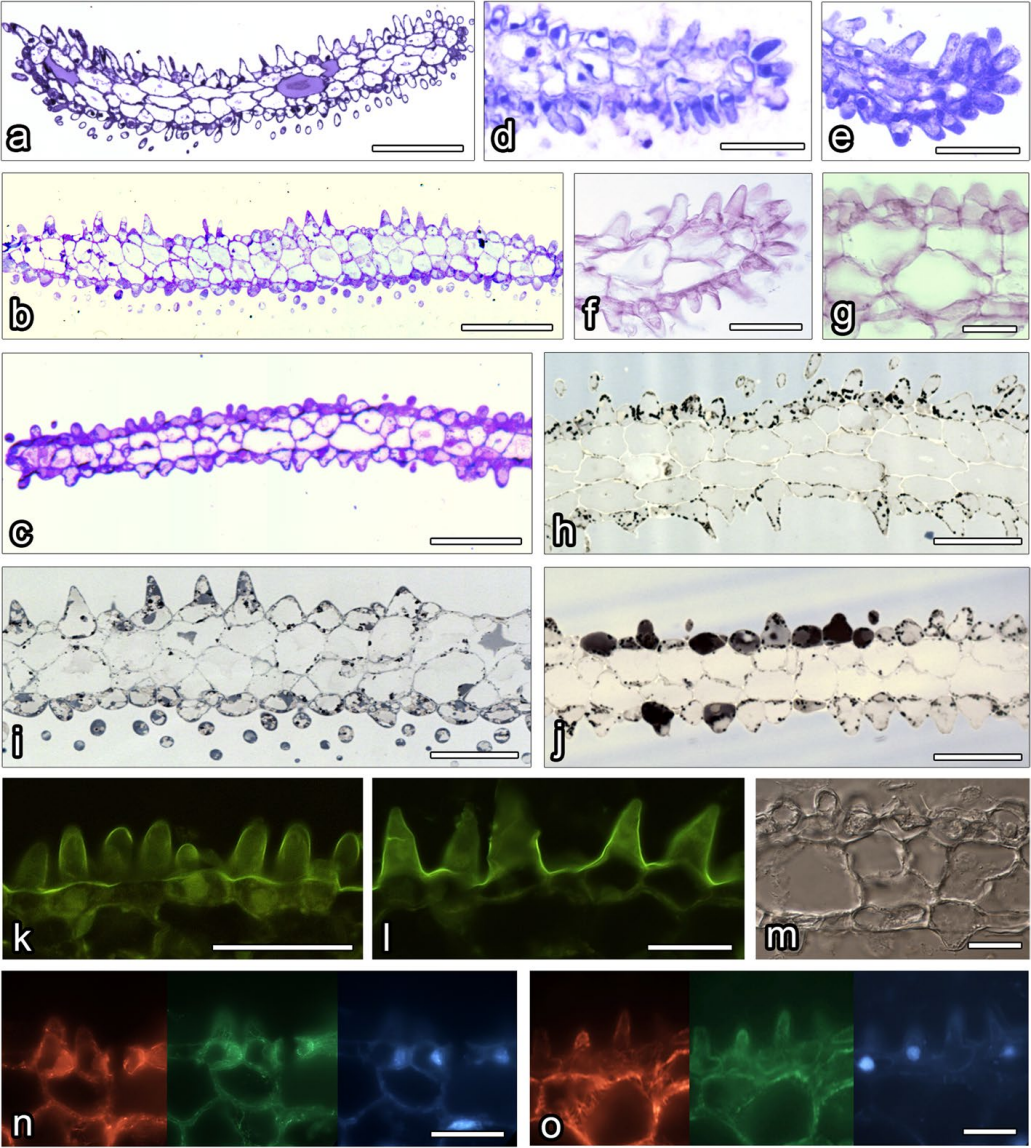
Histochemical features of lip of *L. calodictyon* (**a**, **d**, **f**, **h**, **k**, **n**), *L. saltatrix* (**b**, **i**, **l**, **o**) and *L. tentaculata* (**c**, **e**, **g**, **j**, **m**). **a** – **c** transverse section (TBO), **d** and e proteins in the epidermal cells (CBB), **f** and **g** absence of starch grains in epidermal and subepidermal cells (PAS), **h** – **j** lipid droplets in epidermal cells (SBB), **k** and **l** smooth and intact cuticle (Auramine O), m absence of dihydroxyphenols inclusions in cytoplasm of epidermal cells (FeCl_3_), **n** and **o** dense network of microtubules (red fluorescence) and microfilaments (green fluorescence) chromatin of nuclei of epidermal cells (blue staining). Bars: **a**, **b**, and **c** – 100 µm; **d**, **e**, **f**, **h**, **i**, **j**, and **k**,—50 µm; **l** – 20 µm; **g**, **m**, **n**, and **o** – 10 µm

The morphology of the lip was the most diverse feature, and it varied in shape between the species. However, it was always hinged on the claw and never trilobed, in contrast to most *Lepanthes* species. The surface of the lip of each examined species was covered by unicellular papillae, which were conical with rounded tips (Fig. 6a–c). No ornamentation was observed on the cuticle of the labellar papillae. The only difference between species was the presence of longer and shorter forms of the papillae on the margin of the *L*. *calodictyon* lip. The intense colouration of the lip was observed when stained with neutral red and methylene blue solutions.

The whole surface of the *L*. *calodictyon* lip was covered by unicellular, bottle-shaped papillae, which were formed by epidermal cells. Papillae were covered by a smooth cuticle and no secreted substances were observed (Fig. 1b, c). Only a few lengthened trichomes were observed on the corner of the lip (Fig. 1b). The cuticle of the trichomes was smooth, and no secreted substances were observed.

The ray-like lip of *L*. *saltatrix* was built by regular epidermal cell, forming papillae. The entire area of the lip (Fig. 2b, c) was covered by this kind of cell, whereas longer unicellular trichomes were observed at the corner of the edges (Fig. 2b). The cuticle of the papillae and trichomes was smooth.

**Fig. 7:**
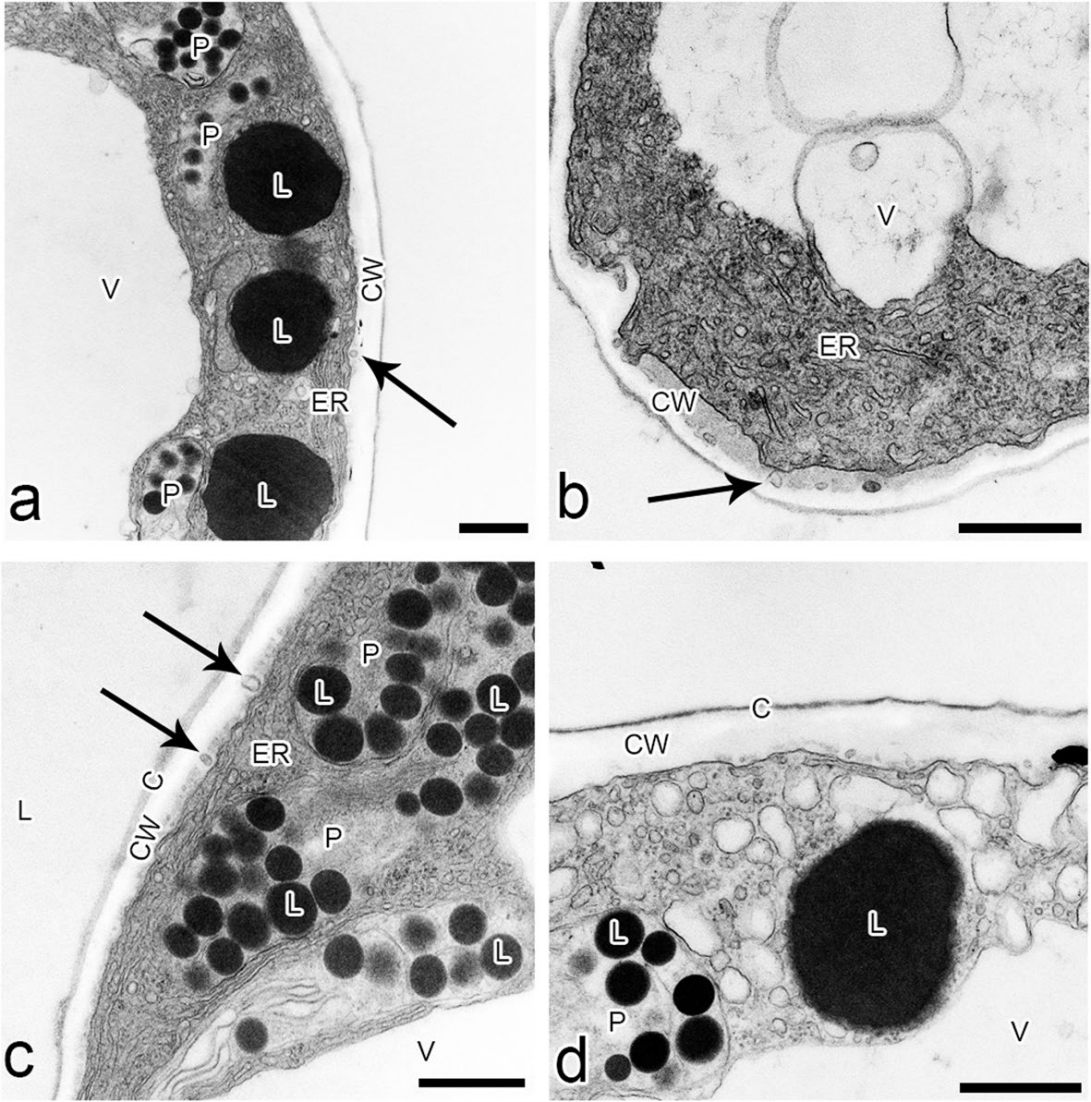
Ultrastructural features of *L. calodictyon* lip. Some of vesicles connected with plasmalemma (**a** and **b**, black arrow). Cuticle is smooth without abruptions (**a**–**d**). In peripheral dense cytoplasm visible abundant ER (**a**–**c**), accompanied with plastids with numerous plastoglobuli and internal membranes (**a**–**d**), lipid droplets (**a**, **d**), vesicles (**b**, **d**) while the centre is occupied with large vacuole (**a**–**d**). c cuticle, cw cell wall, ER endoplasmic reticulum, l lipid droplets, p plastid, v vesicles, v vacuole. Bars: 500 nm

**Fig. 8:**
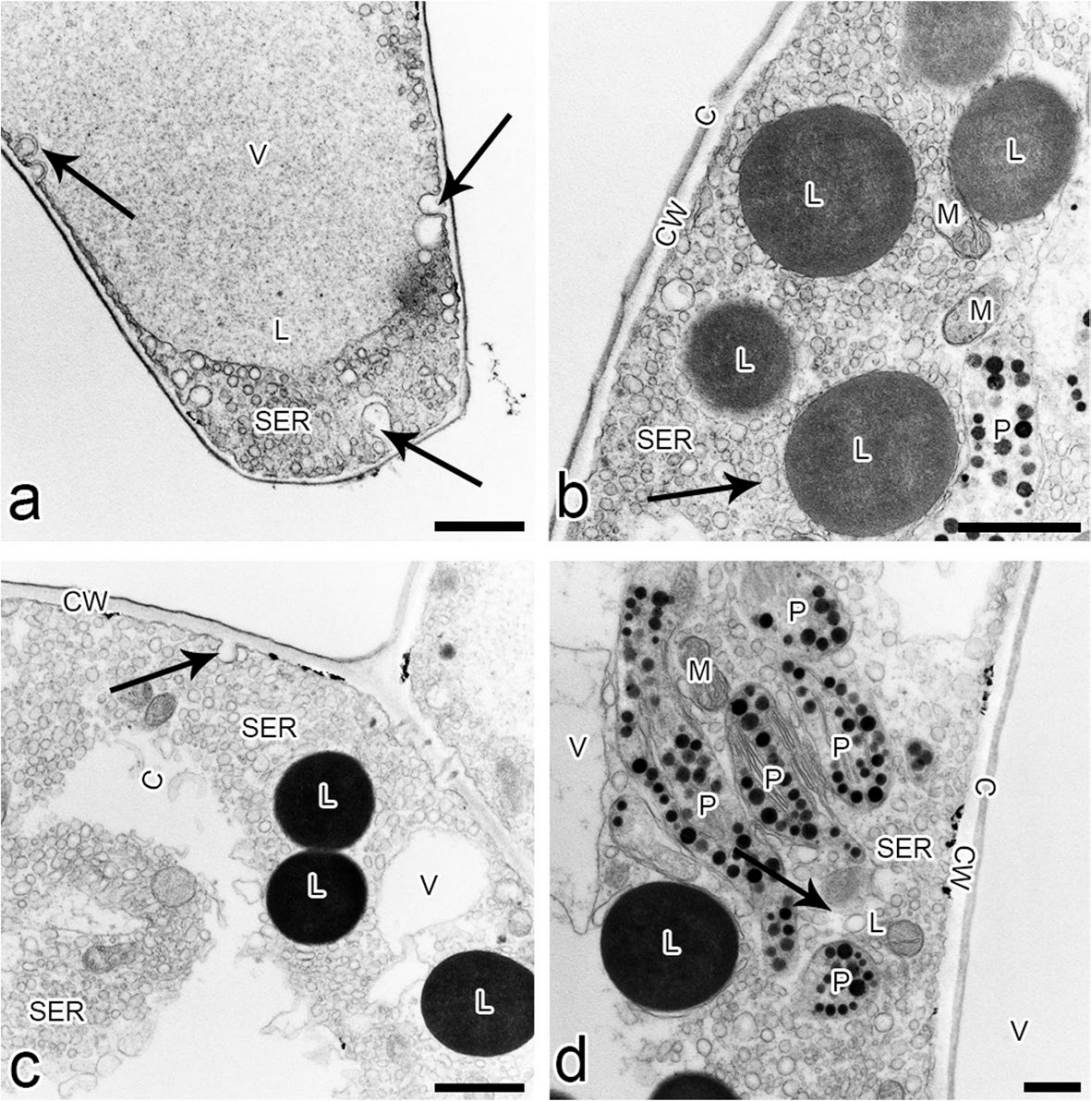
Ultrastructural features of *L. saltatrix* lip. Dense, peripheral cytoplasm of trichomes with abundant SER and vesicles (**a**–**d**), lipid droplets (**b**–**d**) and plastids with plastoglobuli and internal membranes (**b**, **d**). Note the invaginations of plasmalemma (**a** – **c**). Cuticle without disruptions (**a**–**d**). c cuticle, cw cell wall, SER smooth endoplasmic reticulum, l lipid droplets, p plastid, v vesicles, v vacuole. Bars: **a** and **c**—1 µm; **b** and **d** – 500 nm

**Fig. 9:**
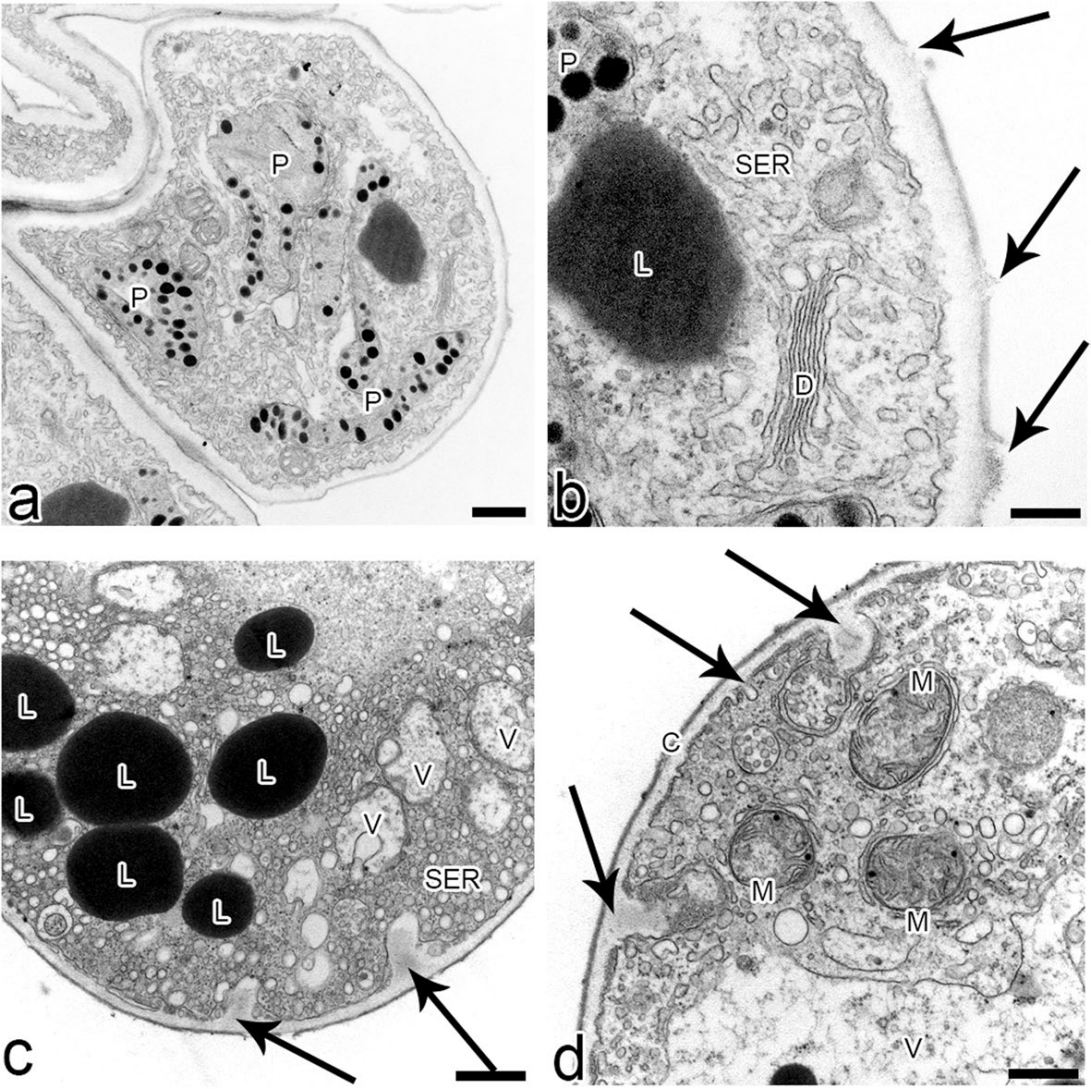
Ultrastructural features of *L. tenatculata* lip. Epidermal cells with numerous SER, vesicles, well developed dictyosomes and mitochondria (**a**–**d**). Plastids with plastoglobuli (**a**). Vesicles building into plasmalemma (**b**–**d**) and some lipid droplets (**c**) observed in dense cytoplasm. Cuticle with visible abruptions (**b**). c cuticle, cw cell wall, d dictyosomes, ER endoplasmic reticulum, l lipid droplets, m mitochondria, p plastid, v vesicles, v vacuole. Bars: **a**, **c**, and **d** – 500 nm; **b** – 200 nm

The ray-like lip of *L*. *tentaculata* was built by regular epidermal cells, forming papillae. The whole middle area of the lip (Fig. 3b) was covered by this kind of cell, and longer unicellular trichomes on the edges were observed. The cuticle of the papillae and trichomes was smooth; however, no secretion particles were presented (Fig. 3c). The cuticle was smooth and intact when stained with Auramin O (Fig. 6k, l). Unlike petal examination, only a few small abruptions of the cuticle were observed on the *L*. *tentaculata* lip (Fig. 9b, black arrows). CBB staining showed that the papillae were generally rich in proteins in all species (Fig. 6d, e), but the longer papillae found on the margin of *L*. *calodictyon* were more intensely stained compared to the shorter papillae (Fig. 6d). Treatment with SBB indicated high amounts of lipids in all examined species (Fig. 6h–j). The PAS for starch grains (Fig. 6f, g) and staining for dihydroxyphenoles were negative (Fig. 6m). The TEM studies revealed the presence of features associated with secretory activity, but no traces of secretion were detected on the surface of the papillae. The main part of the papillae of the lip was occupied by a vacuole, and the cellular structures were crowded along the perimeter (Fig. 5c, f, i). The cytoplasm was dense with a rough or smooth endoplasmic reticulum (Figs. 7–9), mitochondria with well-developed cristae (Figs. 8b and 9d), numerous vacuoles and dictyosomes (Figs. 7–9), plastids with plastoglobuli (Figs. 7a, c, d, 8d and 9a). Small vesicles were observed between irregular plasmalemma and the cell wall. Some of the vesicles were connected to plasmalemma (Figs. 7a, 8a and 9c, d; black arrows). The cytoskeleton of the lip was well developed in all three species compared to the trichomes of the petals (Fig. 6n, o).

## Discussion

Floral features potentially associated with pseudocopulatory pollination include three dimensional flower forms, contrastive colouration, the presence of narrow, unicellular trichomes with frequently pointed tips, and reflective surfaces. Various combinations of these traits have been reported in orchids, including *Mormolyca* Fenzl. [13] , representatives of *Ophrys* L. [3, 50] and *Trigonidium* Lindl. [51, 63], and other angiosperms, such as *Utricularia* L. [43]. The flowers of all three examined species exhibit characteristics associated with fly pollination orchids, such as colours, a motile lip hinged to the claw and the presence of osmophores, which lure pollinators, as observed in *Bulbophyllum* Thouars [29]. Anatomical investigations of *L*. *calodictyon*, *L*. *saltatrix* and* L*. *tentaculata* confirmed the presence of osmophores on the surface of the lip and petals. The cytoplasm of the labellar and petal cells of examined taxa is dense with a large amount of organelles, including numerous mitochondria, lipid bodies, vacuoles and vesicles. The plastoglobuli are surrounded by a well-developed endoplasmic reticulum and cytoskeleton network. The anatomy of the cells suggests high metabolic activity in the cell, such as that in osmophoric tissues [29, 46, 58].

Osmophores were observed on the epidermal and subepidermal layers (e.g. [2, 12, 42, 56, 62]). The subsecretory parenchyma is characterised by the presence of numerous starch grains, and they provide energy for secretory activity through polysaccharide hydrolysis. In osmophores, starch grains occur frequently, but not exclusively, with lipid bodies [62]. Cells in the *L*. *calodictyon* group lack starch grains, but numerous lipid bodies were observed. Osmophores without starch have been previously reported among various orchid species, including *Anacamptis pyramidalis f*. *fumeuxiana* (L.) Rich. [31], *Bulbophyllum wendlandlianum* (Kraenzl) Dammer [28], *Cyclopogon elatus* (Sw.) Schltr. [64], *Cypripedium* L. [59] and *Gymnadenia conopsea* (L.) R.Br [55]. In the orchid *Grobya amherstiae* Lindl. [40] and carnivorous plants nsarin e*Utricularia cornigera* Studnicka and *U*. *nelumbifolia* Gardner, only the epidermis has been identified as highly active physiologically. The conclusion about the physiological condition was made based on the ultrastructure and histochemistry.

In the case of the *L*. *calodictyon* group, the papillae on the surface of the lip and petals can emit the fragrance detectable by pollinators. The presence of plastids with plastoglobuli suggests a site for volatile compound synthesis, as they were found in osmophores and nectaries [54, 55]. The proximity of the endoplasmic reticulum to plastoglobuli is often considered indicative of fragrance production. The fragrant compounds may be produced in plastoglobuli and transported via the endoplasmic reticulum to the plasmalemma or as lipophilic substances directly in the cytoplasm [29, 31, 39, 52, 54]. This assumption concerning fragrance emission in the flowers of the *L*. *calodictyon* group is supported by anatomical evidence. The endoplasmic reticulum is connected to the plasmalemma, as observed in other orchid genera, including *Restepia* [46] and *Epipactis* Zinn [28]. Ultrastructural and histochemical analyses revealed lipid bodies in the petal and lip cells of all studied species, supporting the potential for fragrance production. The possibility of fragrance synthesis was also suggested by cells containing large lipid bodies and the presence of elongated plastids surrounded by a well-developed endoplasmic reticulum [29, 33], as observed in the *L*. *calodictyon* group. The lipids in the cytoplasm have previously been interpreted as indicators of fragrance production, but not necessarily to a fragrance noticeable to humans [11, 46]. The lack of a detectable fragrance in the *L*. *calodictyon* group does not rule out its secretion by flower elements. Similarly, in the genus *Specklinia*, in which ultrastructural analyses have indicated the flower's ability to secrete a scent attracting *Drosophila*, the scent is undetectable by humans [27].

Starch accumulation is typical of osmophore cells [2] and may serve as an energy source for fragrance production via mitochondrial activity. However, histochemical studies and ultrathin sections revealed the absence of starch grains in the cytoplasm of* L*. *calodictyon*, *L*. *tentaculata* or *L*. *saltatrix*. However, the presence of numerous plastoglobuli and lipid bodies in the cytoplasm suggests that starch reserves are metabolised prior to anthesis [28]. The transport of secreted substances through the plasmalemma is often facilitated by wall ingrowths. The numerous vesicles and cell wall ingrowths observed in the studied species suggest granulocrine secretion.

The fusion of the vesicles with plasmalemma is interpreted as granulocrine secretion in orchids [31]. The vesicles close to plasmalemma have been found in other orchid genera, including *Anacamptis* [31], *Bulbophyllum* [29], *Epipactis* [28] and *Gymnadenia* [57]. Cuticular ruptures, similar to those found in the present study, have been reported as a method of exudation of substances to the outside of secretory cells [12]. This has also been demonstrated in orchids, including *Stanhopea oculata* (Lodd.) Lindl. [52], *Bulbophyllum* [29] and *Platanthera chlorantha* (Cluster) Rchb. [56], and other plants, including *Passiflora suberosa* L. [20], *Prunus persica* (L.) Batsch [48] and *Orbea variegata* (L.) Haw. [42]. The epidermal disruptions found in all specimens from the *L*. *calodictyon* group seem to support possible granulocrine secretion.

Although no visible residues of secreted substances were observed on the cell surfaces, this does not preclude fragrance production. This can be explained by the periodic production and release of fragrances that leave no detectable trace [62]. Fragrance volatilisation has previously been described in other orchids [11, 53, 55, 62].

In representatives of the *L*. *calodictyon* group, papillae without starch grains, the presence of lipid bodies and plastoglobuli, and no traces of secreted substances have been previously reported, for example, in *B*. *saltatorium*. Furthermore, the anatomy of the papillae in both cases differed from the elaiophores, which have been frequently reported in orchids [38, 41].

## Conclusions

Morphological and anatomical features of the papillae are similar to those observed among orchid taxa. These structures were identified as osmophores. Additionally, the papillae of the *L*. *calodictyon* group were rich in lipids and proteins, which was correlated with their high metabolic activity. Due to the structural similarities, we suspect that the papillae on the surface of the lip and petals of examined taxa could emit a fragrance. Despite voucher documentation of the studied specimens, the possibility of intraspecific variability remains a factor to consider when interpreting the findings.

## Data Availability

The original microphotographs used and/or analysed during the current study are available from the corresponding author on reasonable request. Reference plant material has been deposited in UGDA Herbarium.

## Abbreviations

CBB: Coomasie Brilliant Blue
DAPI: 2-[4-(Aminoiminomethyl)phenyl]-1*H*-Indole-6-carboximidamide hydrochloride
LM: Light microscopy
PAS: Periodic acid-Schiff reaction
PBS: Phosphate buffered saline
PFA: Paraformaldehyde
RER: Rough endoplasmic reticulum
SBB: Sudan Black B
SEM: Scanning electron microscopy
SER: Smooth endoplasmic reticulum
TEM: Transmission electron microscopy
UGDA: Herbarium Universitatis Gedanensis, Wita Stwosza 59, Gdansk
