# Correction: Floral anatomy and ultrastructure of *Lepanthes calodictyon*,* L. saltatrix* and *L. tentaculata* (Orchidaceae)

**DOI:** 10.1186/s12870-025-07717-x

**Published:** 2025-11-13

**Authors:** Max Rykaczewski, Małgorzata Kapusta, Magdalena Narajczyk, Dorota Łuszczek, Emilia Brzezicka, Dariusz L. Szlachetko, Kapusta Małgorzata

**Affiliations:** 1 Laboratory of Biologically Active Compounds, Intercollegiate Faculty of Biotechnology UG & MUG, University of Gdansk, Abrahama 58, Gdansk, 80-307 Poland; 2 Department of Plant Experimental Biology and Biotechnology, University of Gdansk, Wita Stwosza 59, Gdańsk, 80-308 Poland; 3 Laboratory of Electron Microscopy, University of Gdansk, Wita Stwosza 59, Gdansk, 80-308 Poland; 4 Department of Plant Taxonomy and Nature Conservation, University of Gdansk, Wita Stwosza 59, Gdansk, 80-308 Poland

**Correction: BMC Plant Biol 25**,** 1252 (2025)**

**https://doi.org/10.1186/s12870-025-07141-1**

Following publication of the original article [1], the authors identified an error in the capturing of author names. Unfortunately, the given names and family names were switched. The correct names are presented below (given name/family name):

Max Rykaczewski1 , Małgorzata Kapusta2* , Magdalena Narajczyk3 , Dorota Łuszczek3 , Emilia Brzezicka2 and Dariusz L. Szlachetko4

The author group has been updated above and the original article [1] has been corrected.
